# The expression of NLK is functionally associated with colorectal cancers (CRC)

**DOI:** 10.7150/jca.62526

**Published:** 2021-10-17

**Authors:** Xinyan Chen, Yifan Zhou, Yufeng Wan, Tingting Chen, Huaqing Zhu, Xiaowen Cheng

**Affiliations:** 1 Department of Clinical Laboratory, The First Affiliated Hospital of Anhui Medical University, Hefei, Anhui 230022, P.R. China.; 2 Laboratory of Molecular Biology and Department of Biochemistry, Anhui Medical University, Hefei, Anhui 230022, P.R. China.; 3 Department of Pathology, The First Affiliated Hospital of USTC, Division of Life Sciences and Medicine, University of Science and Technology of China, Hefei, Anhui, 230036, China.; 4 Department of Otolaryngology, The Affiliated Chaohu Hospital of Anhui Medical University, Hefei, Anhui 238001, P.R. China.; 5 Department of Pathology, Anhui Medical University, Hefei, Anhui 230032, P.R. China.

**Keywords:** colorectal cancer, NLK, single nucleotide polymorphism, LncRNA XIST, microRNA-92b-3p

## Abstract

The regulatory mechanism of NLK in the carcinomagenesis and progression of colorectal cancer (CRC) remains unclear. Here, we identified a single nucleotide polymorphism (SNP) site of NLK (rs2125846) as a new susceptibility locus for CRC risk located within an intron of the human NLK gene in a Chinese population. NLK downregulation led to a decrease in the ability of proliferation and migration of RKO cells *in vitro*. The proportion of RKO apoptotic cells increased by interfering with the endogenous expression of NLK. We speculate that LncRNA XIST may upregulate NLK expression by downregulating miR-92b-3p, thereby promote the development of CRC. These results provide important information for the identification of novel potential targets for the prevention or treatment of CRC.

## Introduction

Colorectal cancer (CRC) is one of the most common malignant cancers worldwide. CRC treatments, including surgical resection, radiotherapy, and chemoradiotherapy, have improved in recent years. Statistics show that 50% of patients with stage III can be cured by surgery, 20% of patients will survive following adjuvant chemotherapy, and 30% of patients will relapse within 2-3 years. Therefore, only 20% of patients with stage III benefit from chemotherapy, leaving 80% exposed to unnecessary toxicity 1. Moreover, the prognosis of CRC patients is based on their clinicopathological features, mainly focusing on the stage at diagnosis. The five-year overall survival rate is ~90% for the first stage and then decreases to 70%, 58%, and less than 15% for the second, third, and fourth stages, respectively. Many studies have focused on searching for well-known oncogenes or tumor-suppressor genes in CRC. Significant technological developments in the field of molecular biology have been used in clinics to detect gene expression in tumor tissue samples. Owing to the limited predictive value, high cost, and unconventional use of some clinical molecular markers and the lack of clear treatment guidance, selecting the most effective therapy for CRC patients remains a challenge. Therefore, identifying and exploring new molecular indicators is a topic of high interest 2.

Approximately 35% of CRC risk can be attributed to genetic factors 3. Single nucleotide polymorphisms (SNPs) constitute more than 90% of the genetic variation in the human genome. SNPs can lead to abnormal cell differentiation and promote tumor development. In the past 10 years, more than 60 SNPs have been found to be associated with CRC 4. Nemo-like kinase (NLK) is an evolutionarily conserved mitogen-activated serine/threonine protein kinase 5. In 1994, it was first reported that nemo gene mutation decreased the survival of Drosophila and led to abnormal head and eye development. The mammalian nemo homolog was cloned in 1998 and named NLK 6. Extensive evidence shows that abnormal NLK expression is closely related to the carcinomagenesis and progression of human cancers. It is highly expressed in laryngeal carcinoma, lung cancer, and cervical squamous cell carcinoma 7-9 but remains at low levels in non-small cell lung cancer, breast cancer, and ovarian cancer 10-12. However, the contribution of SNPs to the expression of NLK in relation to the risk to CRC is much less clear. Previous studies analyzing more than 1000 samples and nearly 400 SNP sites in 67 mitotic kinases found that rs2125846 in the NLK gene is associated with an increased risk of ovarian cancer 13. In this case-control study, we analyzed the relationship between this NLK SNP and the risk of CRC in a Chinese population to provide a reference for the prevention and treatment of CRC in high-risk groups. However, the expression change of NLK in CRC is still controversial. Li et al analyzed the protein expression of NLK in CRC tissues by immunohistochemical (IHC) analysis and found that its expression was increased in colorectal cancer tissues compared with adjacent normal tissues 14,15. Han et al found that the mRNA level of NLK in CRC tissues was lower than that in adjacent tissues by RT-PCR 16. In this study, we detected the expression of NLK in CRC tissues and observed that changes in NLK levels affected the biological behavior of CRC cells at the cellular level.

The genome sequencing project has revealed that more than 90% of genomes are transcribed into non-coding RNA (ncRNA), and only 2% of the human genome consists of protein-coding genes 17. With the recent development of functional genomics, ncRNA has become a primary focus of gene regulation research. Long-stranded non-coding RNA (LncRNA) and microRNA (miRNA) are the two most intensively studied ncRNAs 18. Increasing evidence shows that the occurrence and progression of many diseases, including cancer, are related to an imbalance in the regulation of miRNAs and LncRNAs 19,20. In 2009, Seitz et al 21 proposed that most RNA transcripts with miRNA response elements (ncRNAs) can act as competitive inhibitors of miRNA. RNAs involved in this process are termed miRNA sponges, and they regulate the expression and function of miRNA by competing with endogenous mRNA for miRNA binding sites. In 2011, Salmena et al. 22 proposed the competitive endogenous RNA (ceRNA) hypothesis. They speculated that ncRNAs, especially LncRNAs, can act as endogenous miRNA sponges to inhibit miRNA function, thus affecting diverse miRNA targets. This interaction can decrease the level and activity of miRNAs. In recent years, ncRNAs, including microRNAs and LncRNAs, have been increasingly suggested as a new class of clinical biomarkers and potential targets for cancer therapy 23.

LncRNAs participate in the development of CRC by coordinating with miRNAs and protein-coding mRNAs. LncRNAs modify the expressions of targeted genes by competinging with miRNAs for the binding sites on the mRNAs. Recent studies have shown that regulatory networks formed by the LncRNA/miRNA/mRNA interactions regulate the initiation and development of CRC 24. miRNA-92b-3p (miR-92b-3p) directly targets NLK in oral squamous cell carcinoma 25 and glioma 26, and lncRNA XIST directly acts on miR-92b-3p in hepatoma cells 27. Therefore, via analyzing the effect of NLK on the CRC cells, we further explored the roles of LncRNAs and miRNAs in regulating the expression of NLK. More specifically, we addressed the relationships among XIST, miR-92b-3p, and NLK in CRC first by investigating the expressions of XIST and miR-92b-3p in the CRC and normal colorectal tissues, and by analyzing the effect of the XIST/miRNA-92b-3p/NLK signaling axis on the carcinomagenesis and progression of CRC, using a dual luciferase reporter system and by silencing XIST and miR-92b-3p.

In this study, we explored the possible roles of NLK in the carcinomagenesis and progression of CRC from clinical perspectives with hope to provide meaningful implications for searching the markers for early diagnosis as well as drug targets for CRC treatment.

## Materials and methods

### Patients and samples

147 CRC cases and 150 healthy subjects were enrolled in the present study. Healthy participants who matched the age and sex of the case group were randomly selected as the control group. Thirty-eight fresh CRC tissue pairs (tumor and adjacent normal tissues) were obtained by surgical resection from the First Affiliated Hospital of Anhui Medical University and immediately stored in RNAlater at -80 °C. All cases were diagnosed by histopathology, and patients with preoperative radiotherapy and chemotherapy or with autoimmune diseases such as diabetes and nephropathy were excluded. The tissue wax blocks, and sections of CRC were obtained from the Department of Pathology of the First Affiliated Hospital of Anhui Medical University and confirmed via histopathological diagnosis by pathologists. Informed consent was obtained from each enrolled patient. The study protocols were approved by the Ethics Committee of Anhui Medical University.

### DNA extraction

DNA was extracted from peripheral blood leukocytes according to the manufacturer's instructions of the DNA extraction kit (Baiao Company, Shanghai). The content and purity of DNA were detected using a NanoDrop One/OneC micronucleic acid protein concentration analyzer (Thermo, USA). The ratio of the absorbance at 260 and 290 nm was between 1.8 and 2.0.

### SNP genotyping

The high-resolution melting (HRM) method was used to determine the SNP genotypes. HRM statistics were collected from the Roche LightCycler96 fluorescence quantitative PCR instrument. The primers used were as follows: NLK-SNP-F, GCTCTAACTTCTAATGGAAGTC and NLK-SNP-R, CAGATCCATACATGAGAAACAC.

### Immunohistochemistry (IHC)

Tissue sections were deparaffinized, rehydrated, and heated using citrate antigen repair solution under high pressure for antigen retrieval. Then, the slides were incubated with 3% hydrogen peroxide at room temperature to block endogenous peroxidase activity and subsequently incubated with the appropriate amount of a polyclonal anti-NLK antibody (Cell Signaling Technology, Beijing) for 3 hours at room temperature. The sides were washed three times with PBS, followed by incubation with the second antibody and and treated with DAB for 5 minutes. The slides were examined under a microscope, and the results were analyzed with Image J software to calculate the positive IHC signals.

### TdT-mediated dUTP Nick-End Labeling (TUNEL)

A one-step TUNEL apoptosis detection kit (blue skies, Shanghai) was used to determine the rate of apoptosis in CRC tissues and adjacent normal tissues. After dewaxing in water, 20 μg/ml of DNase-free proteinase K was added for 30 minutes. The TUNEL detection solution (fluorescence labeling solution:TdT enzyme=9:1) was prepared and incubated with tissue sections for 30 minutes. The slides were washed three times with PBS, and DAPI dye solution was added for 3 minutes. After washing with PBS three times, an anti-fluorescence quencher was added dropwise. The slides were mounted and later observed under a microscope.

### Cell culture and transfection

The human CRC cell line RKO was purchased from the American Type Culture Collection (USA). It was then cultured in Dulbecco's Modified Eagle Medium (Hyclone, Beijing, China) with 10% fetal bovine serum (FBS) and incubated in a humidified incubator with 5% CO2 at 37 °C. The NLK overexpression lentivirus vector NLK-LV5 (EF-1aF/GFP&Puro) and NLK interference lentivirus vector NLK-shRNA- LV3 (H1/GFP&Puro) were obtained from Shanghai Jima Pharmaceutical Technology Co. Ltd. For cell transfections, RKO cells were seeded into a 12-well plate, cultured overnight, and transfected with an appropriate amount of lentivirus containing NLK-LV5, negative control (LV5NC), NLK-shRNA-LV3, or negative control (LV3NC). The medium was replaced with fresh medium after 12 hours. The state of the cells and the proportion of GFP expression were observed daily. All transfects were tested regularly by qRT-PCR and western blot to ensure the efficiency of overexpression or knockdown. The target sequences of NLK shRNA were as follows: NLK-KD, 5'-GGATAGACCTATTGGATATGG-3' and LV3NC, 5'-TTCTCCGAACGTGTCACGT-3'.

### Cell proliferation assay

The proliferation rate of RKO cells was measured with the Cell Counting Kit-8 (CCK-8; 7sea, Shanghai) assay and colony formation assay. For the cell count assay, control and transfected cells were cultured in a 96-well plate (2000 cells/well) and incubated at 37 °C in 5% CO2. Triplicate wells were measured in each group. After adding 10 μl CCK-8 solution to each well, the plate was incubated for 2 hours. Then, the spectrophotometric absorbance was measured at a wavelength of 450 nm using a microplate reader (Thermo Scientific, USA) for each sample. For the colony formation assay, control and transfected cells were seeded into a six-well plate (400 cells/well) and incubated at 37 °C in 5% CO2 for 8-10 days. Triplicate wells were measured in each group. Cell culture was terminated when visible clones were observed, and then the colonies were stained with crystal violet. The colonies were carefully washed with ddH2O three times, and images were taken after drying. The number of colonies was counted three times, the average value was obtained, and the number of clones was compared among the groups.

### Cell migration assay

The cell migration assay was performed in a Transwell chamber (8-μm pore size; CORNING). 5×10^4^ cells in 100 μl serum-free DMEM was added to the top chamber, and 600 μl of medium containing 10% FBS were placed into the bottom chamber as an attractant. The non-migrated cells were removed from the top chamber after incubation for 24 h. The chambers were then fixed with a 4% methanol solution, stained with crystal violet, and photographed.

### Flow cytometry detection

Flow cytometry was used to detect the apoptosis and cell cycle phase of CRC cells. Annexin V-PE and 7-ADD double staining (BD Biosciences, America) was performed in accordance with the manufacturer's instructions to detect cell apoptosis. Then, 200 μl 1X Annexin V Binding Buffer was added to the solution, and the cells were analyzed using a flow cytometer. For the cell cycle assay, RNase A and PI staining solutions were added to collected cells in accordance with the supplier's instructions (BeiBo, Shanghai). Then, the cell cycle was detected using a flow cytometer.

### Western blot

Briefly, the harvested cells were washed with PBS and lysed with 1 X SDS lysis buffer. The BCA method was used to measure the protein concentration. Samples were separated by 10% SDS-PAGE and transferred to the PVDF membrane. The membranes were blocked with 5% fat-free milk in PBS, 0.1% Tween 20 for 2 hours at room temperature, followed by overnight incubation with the primary antibody at 4 °C. Next, the secondary antibody was incubated for 1 hour at room temperature, and the protein bands were detected using the ECL reaction solution.

### Quantitative real-time polymerase chain reaction (qRT-PCR)

Total RNA was extracted from cultured cells using a total RNA extraction kit (TIANGEN, Beijing) in accordance with the manufacturer's instructions. Then, cDNA synthesis was performed using a reverse transcription kit (TAKARA, Dalian) following the manufacturer's instructions. A fluorescent quantitative PCR kit (TAKARA, Dalian) was used for qPCR analysis. The relative gene expression level was calculated by the 2ΔCt method. All experiments were repeated three times, and GAPDH was used as the internal control. The following primers were used: NLK: F: 5'-TATCGGGCTCCAGAAATCCT-3', and R: 5'-GTGTGCCCAACAGATCCGT-3'; LncRNA XIST: F: 5'-GAGGCAAGATGGATGATAGCAG-3', and R: 5'-ACAATCACGCAAAGCTCCTAAC-3'; miR-22-3p: 5'-CTGCTATTGCACTCGTCCC-3'; U6: F: 5'-CTCGCTTCGGCAGCACA-3', and R: 5'-AACGCTTCACGAATTTGCGT-3'.

### Dual-luciferase reporter gene assay

The 293T cells were cultured for co-transfections. The XIST and NLK 3′-UTR regions, containing potential miR-92b-3p binding sites, were predicted using TargetScan version 7.11. The predicted 3′-UTR fragments were amplified by PCR. Mutants were then constructed by introducing point mutations into the seed binding site for miR-92b-3p. The wild type and mutant fragments (wt-Luc-XIST and wt-Luc-NLK, and mu-Luc-XIST and mu-Luc-NLK) were subcloned into the psiCHECK2 vector (Promega Corporation, United States), downstream of the renilla luciferase gene. The vector also contains the firefly luciferase gene. Cells were seeded in 24-well plates and cotransfected with wild-type or mutated luciferase constructs along with miR-92b-3p mimics, miR-92b-3p inhibitors, or controls. The Dual Luciferase Reporter Assay System (Promega) was used 48 h after transfection following the manufacturer's protocol. The relative luciferase activity was calculated using the ratio of firefly luciferase activity to renilla luciferase activity.

### Statistical analysis

SPSS20.0 software was used for analysis. The results were subjected to one-way ANOVA and parametric t-testing and were expressed as the mean ± SD. An unconditional logistic regression model was used to analyze the association between different genetic models and CRC, and the odds ratio (OR) and 95% confidence interval (95%CI) were calculated. The AA genotype represents the homozygous wild type, the AG genotype represents the heterozygous mutation, and the GG genotype represents the homozygous mutation. The genetic models used in this study included a codominant genetic model [polymorphic homozygous vs wild-type homozygous, and heterozygous vs wild-type homozygous--(GG/AA and AG/AA)], dominant genetic model [heterozygous + polymorphic homozygous vs wild-type homozygous--(GG+AG)/AA], and recessive genetic model [polymorphic homozygous vs wild-type homozygous + heterozygous --GG/(AA+AG)]. The goal of this analysis using the model referred is to determine risk factors for CRC. A logistic regression model was used to analyze the interaction between multiplicative models. Statistical significance was accepted at a level of P value < 0.05.

## Results

### NLK-rs2125846 (A/G) genotypes correlates with the susceptibility to CRC

This case-control study enrolled 147 CRC patients and 150 healthy subjects. The two groups were comparable and no significant difference in sex or age was found (Table 1).

**Figure 1:**
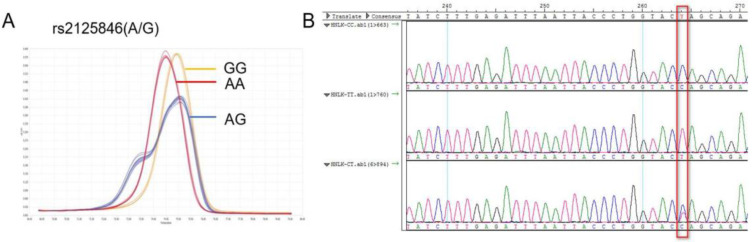
NLK rs2125846 A / G HRM curve (**A**) and sequencing map (**B**).

The NLK gene is located at human chromosome 17p12, rs2125846 is located at the 6^th^ intron (2364A/G) of the NLK gene. An A-G base substitution was identified at position 2364 as follows, GAGAAACACTTTTATGATGTCTGCT[A/G]GTACCAGGGTAATTAAATCTCAA. The reverse mutation was A/G, and the forward mutation was T/C (Figure 1).

Rs2125846 was characterized by AA, AG, and GG genotypes, and its distribution in the control and case groups was in accordance with Hardy-Weinberg genetic balance (P>0.05). In the case group, the AA genotype accounted for 44.1%, AG for 52.4%, and GG for 3.5% of the population, whereas in the control group, the figures were 46.7%, 42.0%, and 11.3% (P<0.05). Therefore, the rs2125846 polymorphism exhibited distinct patterns among the case group and the normal group (Table 2).

Using the co-dominant genetic model, no significant difference in the CRC susceptibility was observed between the AA genotype and the AG genotype groups (*P*>0.05) (OR=0.327, 95%CI: 0.114-0.937). Furthermore, the risk of CRC in patients with the GG genotype was 0.327 times that of the AA genotype. In the dominant genetic model, the AA genotype was considered the control group, and the corresponding P value for GG/AG was 0.654, indicating no significant difference. In the recessive genetic model, compared with the GG genotype, the G-A substitution that results in the AA/AG phenotypes was more closely associated with the onset of CRC (3.528-fold) (Table 3).

The rs2125846 polymorphism was further analyzed in correlation to common clinicopathological features, age of the patients, main location of CRC, drinking history, and family history of the study groups. As shown in Table 4, the polymorphism at this locus was closely related to tumor size, tissue infiltration depth, and drinking history (*P<*0.05) as opposed to tissue differentiation, tissue typing, age, tumor location, distant metastasis, TMN, and family history (*P*>0.05) (Table 4).

### NLK was upregulated in CRC

**Figure 2:**
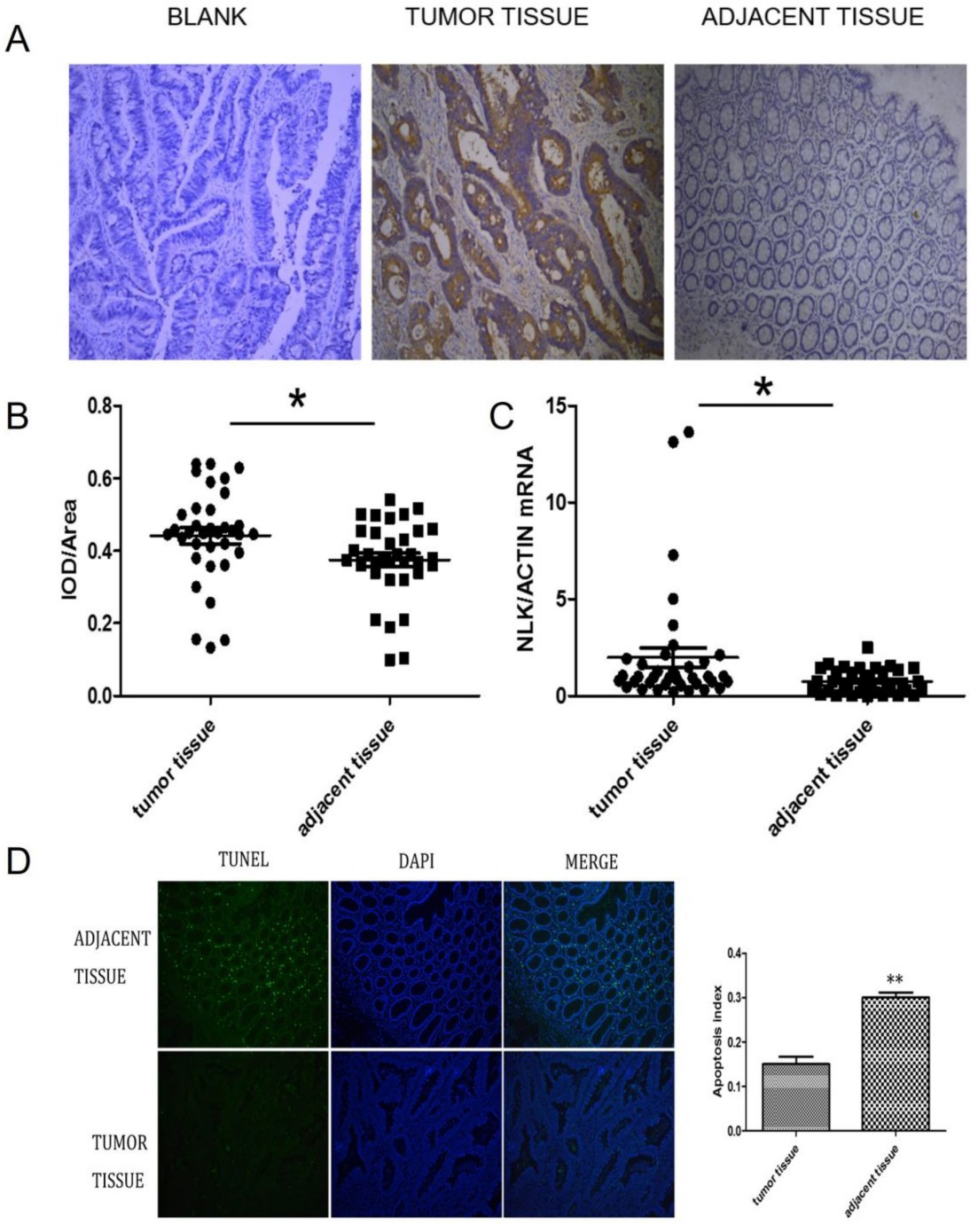
** NLK was upregulated in CRC tissues. (A and B)** Tumor sections stained with DAB, and NLK expression was examined by immunohistochemistry. Magnification, 100×. *P<0.05. **(C)** The mRNA levels of NLK were detected by qRT-PCR in tumor tissues and adjacent tissues. *P<0.05. **(D)** TUNEL assay of CRC and adjacent tissues. Magnification, 100×. **P<0.01. TUNEL, TdT-mediated dUTP Nick-End Labeling.

The expression of NLK were investigated first using IHC. There was a clear increase in the level of NLK in clinical CRC tissues compared with adjacent tissues (Figure 2A), which was also statistically significant (Figure 2B) and was further reflected at transcriptional level when analyzed by qPCR (*P*<0.05) (Figure 2C). The proportion of apoptotic cells, revealed by the TUNEL assay, was doubled (~30%) in the adjacent tissue compared to the one in the CRC tissues (~15%), suggesting an anti-apoptotic role by NLK (Figure 2D).

### NLK downregulation decreased the proliferation, colony forming, and migration abilities of RKO cells

NLK knockdown in the CRC cell lines such as HT-29 and HCT116 affected cell proliferation, colony formation, migration ability, and led to cell cycle arrest at G1/S phase 15, 16. Previous study had designated four main CRC subtypes based on gene expression profiles and summarized as the consensus molecular subtypes (CMS). According to the study, the RKO and HCT116 cell lines were described as CMS1 subtypes, defined by hypermutation, MSI, and strong immune activation, while HT29 cell line was less likely to be appropriate models for CRC tumors. Moreover, result from other study shows that RKO and HT29 are KRAS wild-type cells, while HCT119 cells carry G13D point mutations. And another study shows that RKO and HCT116 cell lines have no TP53 mutation identified DHPLC analysis 28-30. These evidences indicate the differences between RKO cells and other two cell lines. Based on the effect of NLK knockdown on the phenotype of the CRC cell line RKO remains unknown, RKO cells were selected for the following experiments to address this question.

**Figure 3:**
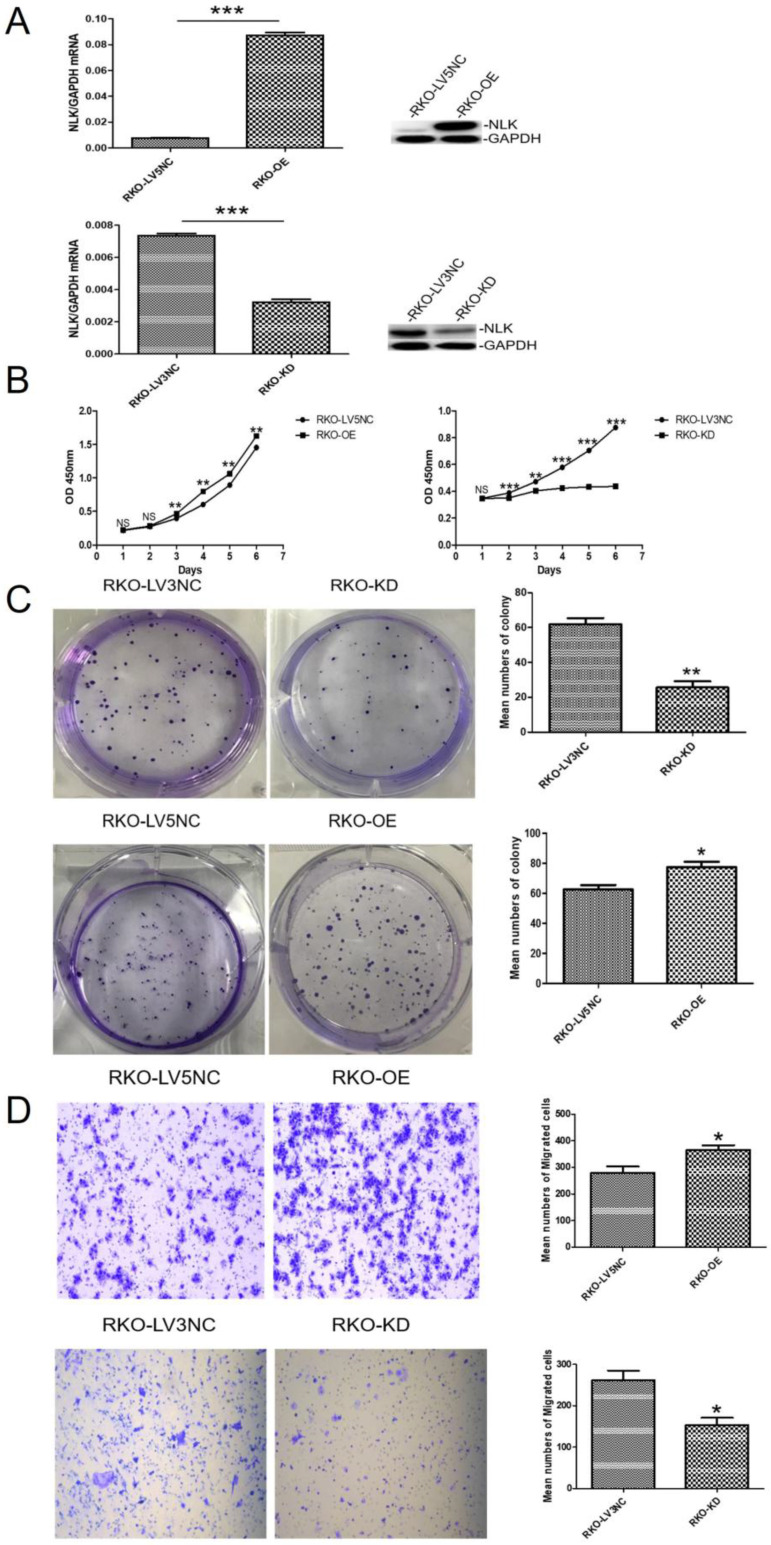
** The expression of NLK affects the proliferation and migration of CRC cells *in vitro*. (A)** Knockdown and overexpression of NLK were confirmed by qRT-PCR (left panel) and western blot (right panel) in RKO cells. ***P<0.001. **(B)** The cell counting assay was performed to measure the proliferation of RKO cells transfected with LV5 compared with those transfected with LV5 NC or shRNA-LV3 and LV3 NC. OD450 values were compared at the indicated time points, **P<0.01, ***P<0.001. **(C)** Representative images and quantification of the colony forming assay in RKO cells transfected with LV5 or LV5 NC and LV3 or LV3 NC, **P<0.01, *P<0.05. **(D)** Transwell assay was performed to determine the effect of NLK overexpression or knockdown on the migration ability of RKO cells. *P<0.05. RKO-OE, RKO cells with overexpression of NLK; RKO-KD, RKO cells with NLK knockdown; q RT-PCR, quantitative real-time polymerase chain reaction.

An ectopic copy of NLK was introduced into RKO cells using a lentivirus vector (NLK-LV5) with the empty vector (LV5 NC) as control. The RNA interference (RNAi) specifically targeting NLK expression was achieved with a lentivirus vector (NLK-shRNA-LV3). The overexpression (OE) lentiviral vector NLK-LV5 (EF-1aF/GFP&Puro) and the RNAi lentiviral vector NLK-shRNA-LV3 (H1/GFP&Puro) were used to infect RKO cells, and then the OE and RNAi stable cell lines were constructed by puromycin selection and flow sorting. The empty backbone vector was used as control for OE and a scrambled shRNA-containing vector as control for RNAi. Both qPCR and Western blot results confirmed the altered expressions of NLK as intended (Figure 3A). The cell proliferation, measured with CCK-8 test and the colony forming assay, appeared significantly suppressed by the RNAi but was moderately upregulated with NLK overexpression. Compared with the corresponding OE control, NLK overexpression leads to an increase in the proliferation rate of RKO cells and in the number of clone formation. Compared with the corresponding RNAi control, the inhibition of NLK expression results in a decrease in the cell proliferation speed of RKO cells and in the number of clone formation (Figure 3B & 3C). Furthermore, the trans-well migration test revealed that NLK RNAi severely compromised the cell migration, in contrast to the promoted migration occurred with NLK overexpression (Figure 3D).

### *NLK* knockdown promoted the RKO cell apoptosis and G1 phase cell cycle arrest of RKO cells

**Figure 4:**
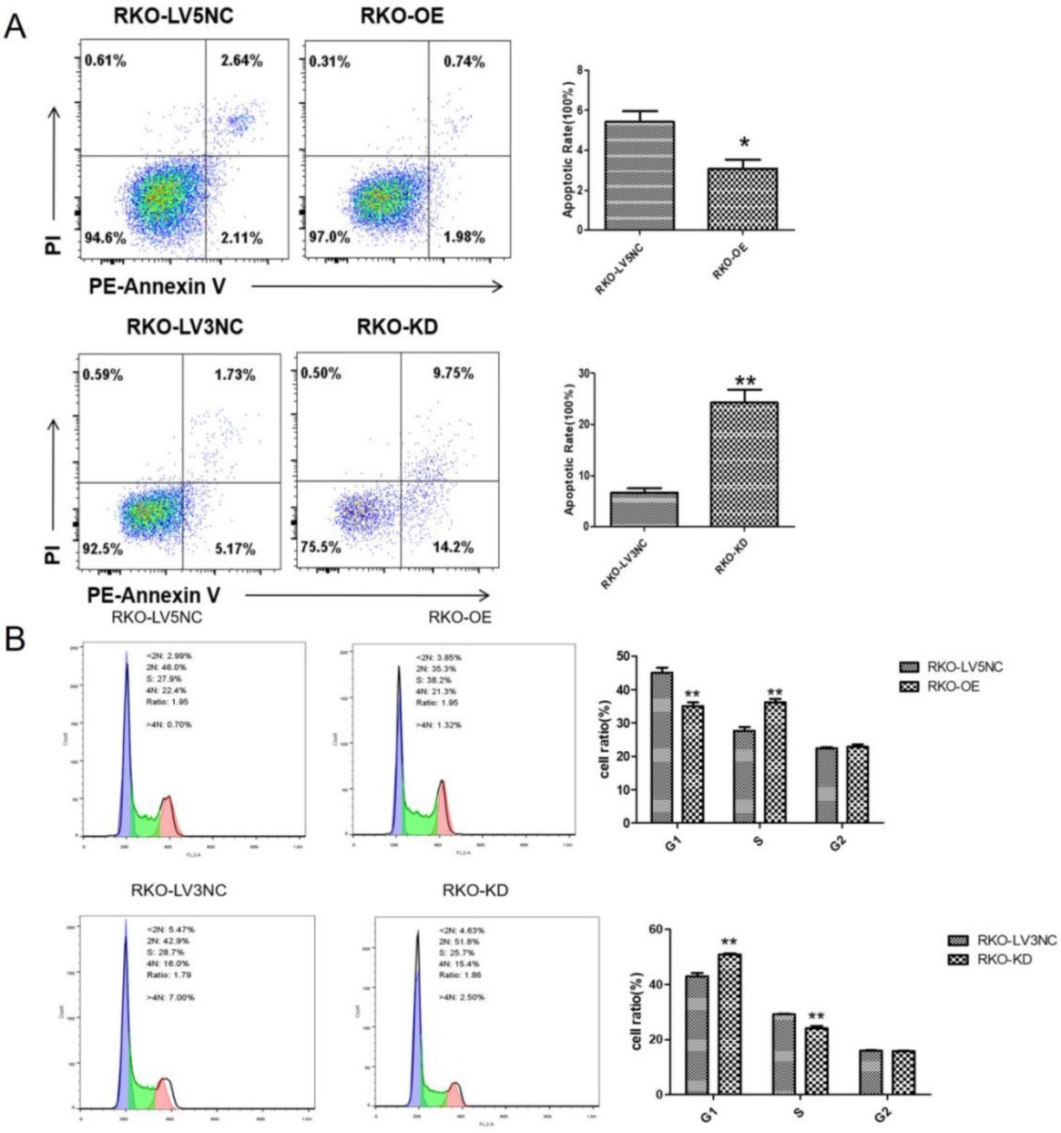
** NLK is required for the cell cycle and apoptosis of RKO cells.** Flow cytometry was used to detect the apoptosis (**A**) and cell cycle (**B**) of RKO cells transfected with LV5 or LV5 NC and shRNA LV3 or LV3 NC. *P<0.05, **P<0.01, **P<0.01. All results were analyzed by FlowJo Software.

Following the clue to the potential link between apoptosis and NLK expression, we used flow cytometry (FACS) to examine the apoptosis and cell cycle of RKO cells upon altering NLK expression. The size of apoptotic cell population was significantly reduced with NLK overexpression (OE) compared with the control, in contrast to the scenario in the NLK-deficient RKO cells where the apoptosis was greatly enhanced (Figure 4A). Specifically, NLK-OE shifted the RKO cells from G1 phase towards S phase. However, this trend was reversed upon NLK RNA*i* (Figure 4B). Therefore, in RKO cells, NLK negatively regulates cell cycle and promotes the apoptosis.

### The correlations between the expressions of XIST, miR-92b-3p and NLK in the context of CRC

**Figure 5:**
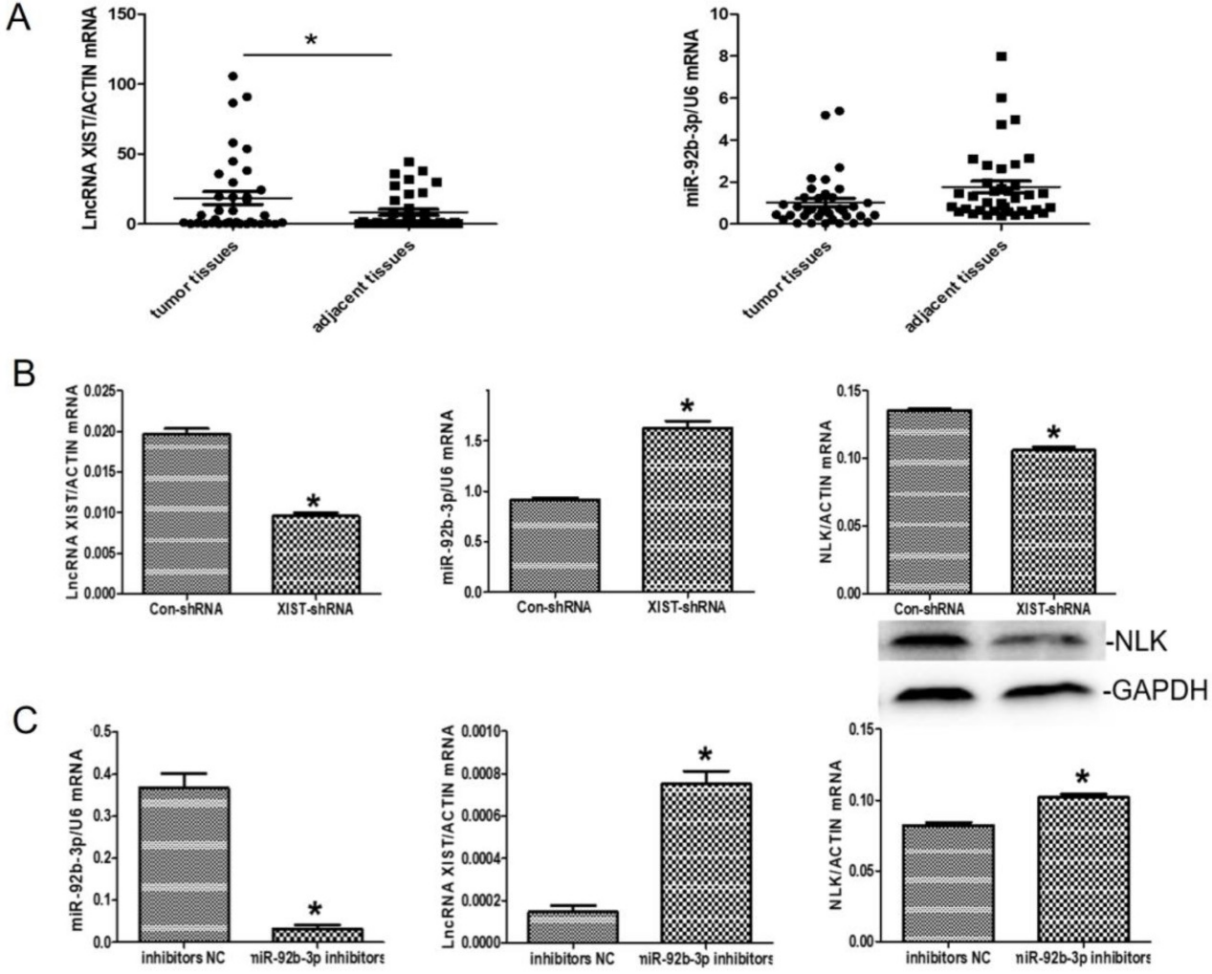
** The expressions of LncRNA XISTXIST, miR-92b-3p, and NLK are interlinked. (A)** The mRNA expression levels of LncRNA XISTXIST and miR-92b-3p were determined using quantitative real-time polymerase chain reaction in tumor and adjacent tissues. *P<0.05. **(B)** qRT-PCR and western blot were used to examine the mRNA and protein expression of miR-92b-3p and NLK following transfection with XIST-shRNA and the negative control. *P<0.05. **(C)** qRT-PCR was used to examine the mRNA and protein expression of LncRNA XISTXIST and NLK following transfection with miR-92b-3p inhibitors and the negative control. *P<0.05.

There was an interesting pattern of differential expression among XIST, miR-92b-3p, and NLK emerging from the CRC clinical samples under the study. Specially, XIST exhibited higher expression in the CRC tissues than in the adjacent tissues whereases the opposite pattern appeared with miR-92b-3p (*P*<0.05) (Figure 5A).

We then explored the functional relevance of this pattern to CRC in RKO cells. In response to XIST downregulation via RNA*i*, the expression of miR-92b-3p was significantly upregulated while NLK expression was suppressed (Figure 5B). When the inhibitor of miR-29b was applied, the expressions of XIST and NLK were both increased (Figure 5C).

### XIST directly binds miR-29b-3p and NLK is a target of miR-29-3p

**Figure 6:**
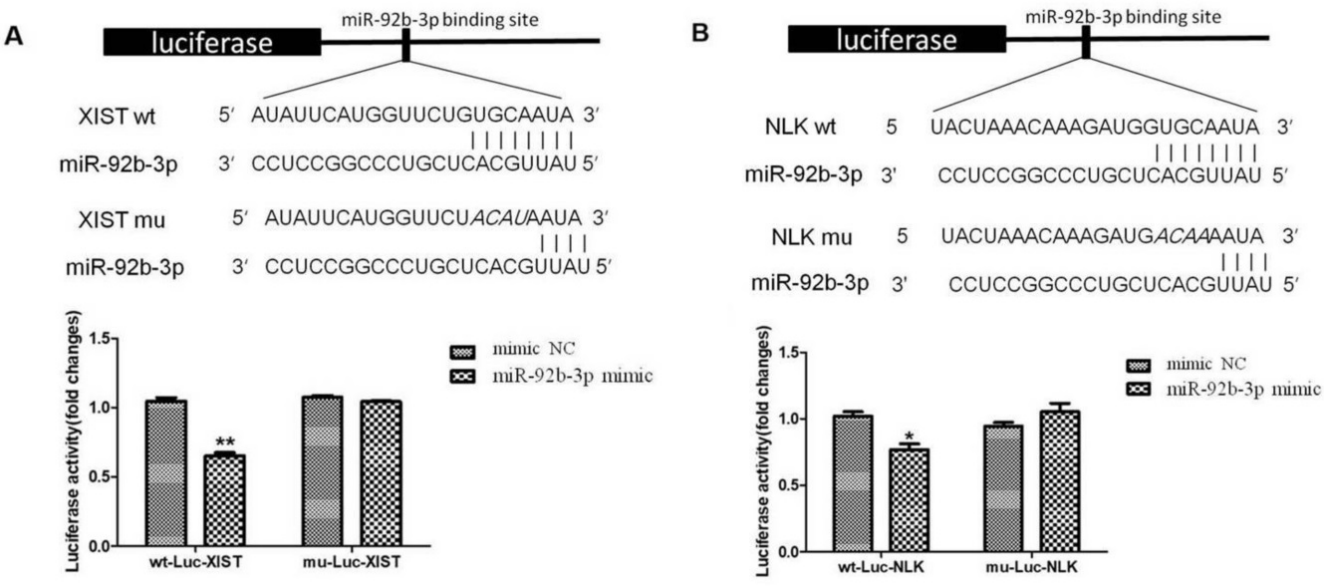
** miR-92b-3p regulates XIST and NLK expressions via directly binding to their mRNAs. (A)** The wild type and mutated miR-92b-3p binding sites in the XIST 3'-UTR are shown in the upper panel. miR-92b-3p mimic and luciferase constructs were co-transfected into 293T cells. Luciferase activity of wild type and mutant Luc XIST are shown in the lower panel. **(B)**The wild type and mutated miR-92b-3p binding sites in the NLK 3'-UTR are shown in the upper panel. Luciferase activity of wild type and mutant Luc NLK are shown in the lower panel. *P* <0.05 vs. control group.

We further tested whether interlinked expressions of XIST and miR-92b-3p or miR-92b-3p and NLK is mediated by direct interactions. We established a luciferase-based reporter system in which was to measure the influence of upstream stimulation on the transcription level of downstream target genes, that is, the influence on the mRNA expression level of target genes. First, miR-92b-3p mimics/NC and Report-XIST-wt/mut were co-transfected into 293T cells, the fluorescent intensity was measured for the expression of ectopic XIST 24 hours post transfection. As shown in Figure 6A, the expression of XIST was significantly suppressed by the presence of miR-92b-3p, which was abolished when specific mutations were introduced into XIST. This suggests a direct binding between XIST and miR-92b-3p.

Next, we focused on the relationship between NLK and miR-92b-3p. Similarly, miR-92b-3p suppressed the expression of NLK, and disturbing the potential binding between two disrupted this regulatory relationship (Figure 6B).

## Discussion

Among all cancers, the incidence of CRC ranks third, and the mortality rate ranks second wordwide. In the last 10 years, the mortality and morbidity of CRC in China have increased 31. Many patients are however only diagnosed when CRC arrives at middle and late stages, and one of key factors for such unfortunate is the lack of specific and sensitive molecular markers for early diagnosis 32.

In the past three decades, an increasing number of SNPs have been confirmed to be associated with CRC risk through extensive genome-wide association studies and candidate gene association studies 33. Particularly, the intronic polymorphic variants can confer susceptibility to diseases as a variety of functional elements are located in introns, ranging from the splicing sites, enhancers, silencers, to cis-acting RNA elements, thereby likely exerting great impact on the target gene expression 34. Among them is the case of NLK, located on human chromosome 17p12. rs2125846 is located in the sixth intron of the NLK gene and is given rise by a A-G base substitution occurs at position 2364 (2364A/G).

Here, we identified a new susceptibility locus, rs2125846, for CRC risk located within an intron of the human NLK gene in a Chinese population. It was statistically related to the tumor size of CRC, the depth of CRC infiltration, and whether the patient had a history of alcohol consumption. We think there are two possible explanations for this association. First, the sample size was insufficient and mainly focused on the Han population in Anhui Province. However, more comprehensive CRC-related risk factors and common clinicopathological features shall be included along with enlarging sample sizes in future work.

NLK has been proposed as a tumor suppressor of therapy interest 35. We found its expression is specifically related to the onset of CRC. Interestingly, it had been shown that NLK overexpression was related to unfavorable clinicopathological parameters such as advanced TNM stage, poor differentiation, lymph node and distant metastasis, and higher recurrence rates 15. Our results confirm that NLK expression affect the cell viability in CRC (Figure 3). We speculate that NLK may play a key role in regulating the carcinomagenesis and progression of CRC. To confirm this prediction, subsequent experiments investigated the changes in the biological behavior of CRC cells following NLK overexpression or silencing.

We showed that NLK expression regulates the proliferation ability & migratory ability of RKO cells (Figure 4). One aspect is linked to the cell cycle progression, in which NLK downregulation led to an accumulation of cells at G1 phase, consistent with the previous report 14. The proportion of apoptotic cells was decreased after overexpression of NLK but increased after NLK downregulation, which was consistent with the results of cell cycle analysis. The cell cycle disorder caused by the cells in G1 causes the initiation of apoptosis procedures, which in turn causes an increase in apoptotic cells. Specifically, cells were arrested in the G1 phase after downregulation of the NLK gene, which led to an increased number of apoptotic cells.

LncRNAs participate in various biological processes by regulating gene expression at the transcriptional, post-transcriptional, and epigenetic levels 36. Recently, LncRNAs have been proposed as miRNAs sponges in the context of diseases and their abnormal expressions are associated with certain cancers 18,37. XIST, the key regulatory factor of mammalian X inactivation, is also linked to various tumors and related medical conditions 38-40. More importantly, XIST was shown to be significantly upregulated in CRC tissues and cell lines, being related to poor prognosis 41. We confirmed here that XIST is upregulated in CRC tissues.

MiR-92b was first reported to be overexpressed in primary brain tumors 42, and then several studies further clarified its carcinogenic effects in glioblastoma 26, non-small cell lung cancer 43, bladder cancer 44, and cholangiocarcinoma 45. In contrast, miR-92b exhibits anti-tumor activity in many gastrointestinal cancers, such as pancreatic cancer 46 and esophageal squamous cell carcinoma 47. The level of miR-92b in circulating exosomes was significantly higher in healthy controls compared with CRC patients, especially in those with TNMII stage 48. In this study, the expression of miR-92b-3p in CRC was found lower than that in the normal adjacent intestinal tissues. However, no clear relationship can be established between mir-92b-3p expression and clinical parameters included, likely due to the limited sample size.

We demonstrated that XIST acts directly on the miR-92b-3p transcript and regulates its expression. In a similar way, miR-92b-3p regulates the expression of NLK. Overall data suggested a possible role ceRNA in CRC cells, in which the XIST/miR-92b-3p/NLK signaling axis could play critical roles, therefore possessing potential therapeutic values.

## Figures and Tables

**Table 1:** Comparison of general information between patient group and normal group

Group	Patient (n=147)	Normal (n=150)	X^2^/t	P value
**Gender [n (%)]**				
male	86 (49.1)	89 (50.9)	0.021	0.884
female	61 (50.0)	61 (50.0)		
**Age**	59.32±11.23	57.24±10.12	1.677	0.095

**Table 2:** The correlation between genotype distribution of NLK rs2125846 and cancer risk

Groups	AA [n (%)]	AG [n (%)]	GG [n (%)]	χ^2^ value	P value
normal	70 (46.7)	63 (42.0)	17 (11.3)	7.79	0.02
patient	63 (44.1)	75 (52.4)	5 (3.5)		

**Table 3:** Genotype distribution and allele frequency of NLK rs2125846 in cases and controls and analysis of unconditional logistic regression model

	Normal [n (%)]	Patient [n(5)]	OR (95%CI)	P value
**Co-dominant genetic model**				
AA	70 (46.7)	63 (44.1)	1	
AG	63 (42.0)	75 (52.4)	1.323 (0.821, 2.132)	0.251
GG	17 (11.3)	5 (3.5)	0.327 (0.114, 0.937)	0.037
**Dominant genetic model**				
AA	70 (46.7)	63 (44.1)	1	
GG+AG	80 (53.3)	80 (55.9)	1.111 (0.701, 1.761)	0.654
**Recessive genetic model**				
GG	17 (11.3)	5 (3.5)	1	
AA+AG	133 (88.7)	138 (96.5)	3.528 (1.266, 9.834)	0.016

**Table 4:** Relationship between clinicopathological features and NLK 2125846 genotype polymorphism

Analytical index	AA	AG	χ^2^	P value
Pathological feature				
**Diameter**				
<5	0 (0.0)	6 (8.2)	-a	0.033
≥5	59 (100.0)	67 (91.8)		
**Tissue differentiation**				
Low	6 (10.7)	15 (20.8)	2.352	0.125
Middle	50 (89.3)	57 (79.2)		
**Tissue typing**				
Ulcerative	45 (76.3)	59 (80.8)	0.404	0.525
Uplift type	14 (23.7)	14 (19.2)		
**Age**				
<50	16 (25.4)	14 (18.7)	1.839	0.399
50-60	15 (23.8)	25 (33.3)		
>60	32 (50.8)	36 (48.0)		
**Tumor site**				
colon	23 (36.9)	32 (42.7)	0.542	0.462
rectum	40 (63.5)	43 (57.3)		
**Infiltration depth**				
T1	4 (6.8)	2 (2.7)	12.824	0.005
T2	11 (18.6)	20 (27.4)		
T3	7 (11.9)	24 (32.9)		
T4	37 (62.7)	27 (37.0)		
**Lymph node metastasis**				
N0	34 (57.6)	37 (50.7)	-a	0.833
N1	18 (30.5)	26 (35.6)		
N2	7 (11.9)	9 (12.3)		
Nx	0 (0.0)	1 (1.4)		
**Distant metastasis**				
M0	59 (93.7)	73 (97.3)	1.116	0.262
M1	4 (6.3)	2 (2.7)		
**TNM Stage**				
I	13 (20.6)	14 (18.7)	2.225	0.531
II	20 (31.7)	25 (33.3)		
III	25 (39.7)	34 (45.3)		
IV	5 (7.9)	2 (2.7)		
**Drinking history**				
No	58 (92.1)	57 (76.0)	6.631	0.012
Yes	5 (7.9)	18 (24.0)		
**Family history**				
No	62 (98.4)	69 (92.0)	2.924	0.087
Yes	1 (1.6)	6 (8.0)		

## Abbreviations

CRC: colorectal cancer
NLK: Nemo-like kinase
SNP: single nucleotide polymorphism
ncRNA: non-coding RNA
LncRNA: Long-stranded non-coding RNA
ceRNA: competitive endogenous RNA
miR: microRNA
IHC: Immunohistochemistry
qRT-PCR: Quantitative real-time polymerase chain reaction
TUNEL: TdT-mediated dUTP Nick-End Labeling
OR: odds ratio
